# Binocular visual field in adults with horizontal strabismus and driving requirements

**DOI:** 10.1038/s41433-022-02319-5

**Published:** 2022-12-02

**Authors:** Fadi Alfaqawi, Jane Young, Stephen B. Kaye

**Affiliations:** 1 Royal Liverpool University Hospital, Liverpool, UK; 2 Department of Eye and Vision Science, Institute of Ageing and Chronic Disease, University of Liverpool, Liverpool, UK

**Keywords:** Ocular motility disorders, Eye diseases

## Abstract

**Objective:**

To determine the horizontal extent of the binocular visual field (BVF) in subjects with horizontal strabismus and whether the BVF falls below the driving standard.

**Methods and analysis:**

Adults with congenital esotropia and infantile exotropia ≤45 Prism Dioptres (PD), and subjects with orthotropia were recruited. The manifest angle of deviation was measured using a simultaneous prism cover test. Monocular Visual Field (MVF) and BVF were measured using the Esterman visual field test. Subjects with diplopia or a manifest angle of strabismus that varied by>8PD or the present of a vertical tropia >8PD were excluded.

**Results:**

Forty-nine subjects were included: 10 with orthotropia, 20 with exotropia and 19 with esotropia. The horizontal extent of BVF (degrees) was significantly smaller in esotropes (122.8 ± 18.8) than in orthotropes (141 ± 6.6) or exotropes (138.3 ± 8.3) (*p* < 0.01). In 6 (31.6%) subjects with an esotropia, the BVF was below the driving standard. The horizontal extent of the visual field (VF) of the amblyopic eyes of patients with esotropia (98.70 degrees, SD 19.76) and exotropia (104.75 degrees, SD16.93) were significantly smaller than those with orthotropia (121.00 degrees SD 3.16) by 22.3 degrees (*p* = 0.004) and 16.25 degrees (*p* = 0.045), respectively. The difference between the summation of MVFs and the BVF was significantly greater in orthotropes (100.6 ± 2.7) than in exotropes (68.9 ± 34.4) and esotropes (74.2 ± 20.7) (*p* < 0.01).

**Conclusion:**

The horizontal extent of BVF is significantly smaller and more variable in adults with congenital esotropia and may fall below the driving standard.

**Strengths and limitations:**

- Largest study on visual fields in subjects with horizontal strabismus including an orthotropic control group who do not have diplopia and who would otherwise meet the driving standard.
- Visual field quality was high but limitation is that visual field repeatability was not undertaken.

**How this study might affect research, practice, or policy:**

The findings of this study would suggest that people with an esotropia should be offered the opportunity to have a binocular visual field test before applying for a driving license. The DVLA may want to consider requesting people with an esotropia to have a binocular visual field test as is a requirement with other ophthalmic conditions such as glaucoma.

## Introduction

The binocular visual field (BVF) is an important component of vision and the performance of different tasks such as driving. If a person wishes to obtain a driving licence but suffers from a condition which is known to affect the visual field such as glaucoma, then to obtain a driving licence, the Driver and Vehicle Licensing Agency (DVLA) states that measurement of the BVF is required [1].

The extent of the BVF, however, may be affected in a variety of conditions. If a person has a strabismus (misalignment of their eyes) but does not have diplopia and has a corrected visual acuity of at least 6/12, then a BVF test is not required for driving a car. It does, however, state that ‘*You must also have an adequate field of vision - your optician can tell you about this and do a test*.’ This, therefore, leaves the onus on the person to find out whether they have an adequate field of vision. It is generally not known, however, if person with strabismus has a reduced BVF and as such there is no reason for an optician to test the BVF for a person with strabismus who may wish to drive. The issue, therefore, is that a person with strabismus would not know if they have a reduced BVF.

Wortham and Greenwald, and Kushner et al., reported a reduced BVF in adults with esotropia (convergent squint) although the actual sizes of the BVF compared with exotropes (divergent squint) and orthotropes (no strabismus) were not provided [2, 3]. Quah and Kaye, noted a reduction in the size of the BVF in children with esotropia and in the ratio of the BVF to sum of the monocular visual fields (MVF) [4]. For adults, however, the actual size and reduction in the extent of the BVF in esotropia using Esterman visual field (the test required for a driving license [1]), has not been provided and therefore, it is not known whether they have reduced BVF which may impact on meeting the driving requirements. Conversely, patients with exotropia are considered to have panoramic view with an expanded BVF [5, 6].

The size of the BVF may depend on the method used. Previous studies have used Goldmann perimetry rather than Esterman visual field and therefore, applying the measured visual field to tasks such as driving is difficult [1]. It is also unclear as to what extent the BVF is related to the size of the strabismus. Some authors have found that the amount of suppression of the visual field depends on the size of the ocular deviation [7, 8, 5, 9], whilst others have not [10, 11].

In the UK, although adults with diplopia must notify DVLA, the DVLA guidance does not, however, include the presence of strabismus as a condition requiring measurement of the BVF. Subjects with strabismus who do not have diplopia, therefore, are not required to demonstrate an intact BVF although they would be expected to have an intact BVF for tasks such as driving. The purpose of this study is to determine the extent of BVF in adults with horizontal strabismus (HS) using Esterman visual field perimetry.

## Methods

Patients with congenital esotropia or infantile exotropia were recruited from the Orthoptic Department of The Royal Liverpool University Hospital. Inclusion criteria were a horizontal tropia of between 0 to 45 dioptres, Visual Acuity (VA) better than 6/60 in the worst eye and 6/12 or better in the fellow eye, and able to perform a visual field test. Exclusion criteria were diplopia, other causes of esotropia or exotropia including paralytic or restrictive strabismus, other ophthalmic disease, previous ophthalmic surgery, VA less than or equal to 6/60 in the worst eye, inability to perform a visual field test as judged by the operator, the presence of nystagmus, manifest angle of strabismus which varied by more than 8PD and manifest vertical strabismus >8PD.

### Angle of strabismus and visual acuity

All the recruited subjects were tested with their refractive error (if present) corrected to provide their best corrected visual acuity (BCVA). The total angle of deviation was measured using an alternate prism cover test at 33 cm and at 6 m and the manifest angle measured using a simultaneous prism and cover test. Best corrected visual acuity (BCVA) was measured monocularly and binocularly at (print size N5 to N48 chart, Keeler) 33 cm and at (logMAR) 6 m. Sensory binocular status was assessed using Bagolini striate lenses without correction of the manifest angle of deviation and Frisby stereoacuity test at near and distance.

### Visual field

The MVF and BVF of each eye (corrected) was measured with a Humphrey Field Analyser (HFA) II (Carl Zeiss Meditec AG, Jena, Germany) using the Esterman visual field output. The subject was asked to fixate a central static fixation target with their head in primary position and asked to press a response button as soon as they were aware of a spot of light (stimulus) appearing on the screen in front of them. Subjects were given opportunity to practice before the test.

### Analysis

For continuous normally distributed data, a Student t test and ANOVA was used. For categorical and or non-parametric data, Chi squared and Krusal-Wallis tests were used. A general linear model was used where the dependent variable followed a normal distribution. A Bonferroni correction was made for multiple tests. The refractive data was transformed into Long’s formalism for analysis [12–14]. For monocular outcomes esotropes and exotropes were grouped into amblyopic (eye with lower BCVA) and fellow eyes, and orthotropes into right and left eyes. To evaluate the contribution of the monocular to binocular visual field, the ratio of the BVF to the sum of the MVF was analysed as previously described [4] and also the difference between the sum of the monocular VFs and the BVF.

### Patient and public involvement

No patient involved.

## Results

Forty-nine subjects were included: 20 with exotropia mean age 40.89 years (SD15.46) mean angle of deviation for distance and near of −21.70PD (13.44) and −22.70PD (14.69) and 19 with esotropia mean age 43.25 years (SD18.96) mean angle of deviation 17.63PD (11.07) and 18.37PD (12.34). Ten subjects with orthotropia mean age 41.90 years (SD13.54), mean stereoacuity of 24.50 seconds (SD 30.86) were included (Table 1). There was no significant difference in the respective ages between the 3 groups (*p* = 0.90, *p* = 0.98).

**Table 1:** Baseline Characteristics Between Groups.

	Esotropes Mean (SD)	Exotropes Mean (SD)	Orthotropes Mean (SD)
Age	43.25 (18.96)	40.89 (15.46)	41.90 (13.54)
BCVA amblyopic	0.34 (0.34)	0.32 (0.33)	−0.11 (0.11) (right eye)
BCVA fellow eye	−0.06 (0.09)	−0.02 (0.14)	−0.12 (0.11) (left eye)
BCVA BEO	−0.06 (0.09)	−0.04 (0.14)	−0.14 (0.07)
SPCT Near	18.37 (12.34)	−22.70 (14.69)	0.00 (0.00)
SPCT Distance	17.63 (11.07)	−21.70 (13.44)	0.00 (0.00)
NVA amblyopic eye	16.21 (14.50)	14.90 (15.93)	5.10 (0.32) (right eye)
NVA fellow eye	5.31 (0.75)	5.42 (1.38)	5.10 (0.32) (left eye)

*BCVA* best corrected visual acuity, *SPCT* simultaneous prism and cover test, *NVA* near visual acuity, *BEO* both eyes open.

### Visual acuity

There were no significant difference in binocular BCVA between esotropes, exotropes and subjects with orthotropia (*p* = 0.10 and 0.10, *p* = 0.09). There was no significant difference in the BCVA of amblyopic eyes (0.02 *p* = 0.99) between patients with esotropia and exotropia, although they were significantly reduced compared to subjects with orthotropia (0.45, *p* < 0.01, 0.43, *p* < 0.01) (Table 1). For the fellow eyes, there was no significant difference between esotropes and subjects with orthotropia (0.06, *p* = 0.08) or between esotropes and exotropes (0.04, *p* = 0.85). There was, however, a significant difference between the BCVA of the fellow eye of patients with exotropes and those with orthotropia (0.10, *p* = 0.028) (Table 1). For the inter-eye difference in BCVA, there was no significant difference between patients with esotropia and exotropia (difference 0.036, *p* = 0.92). There was, however, a significant difference in the inter-eye difference in BCVA between patients with esotropia or exotropia compared to subjects with orthotropia (0.40, *p* = 0.004 and 0.36, *p* = 0.01).

### Visual fields

In orthotropes all false positive (FP) errors were 0% and all false negative (FN) errors were 0% apart from one patient who had 10%. In esotropes all FP errors were 0% apart from one patient had 10% and all FN errors were 0% apart from one patient had 10% FN. In exotropes all FP errors were 0% apart from 5 patients had 10%,10%,11%,20% and 22% and all FN errors were 0% apart 2 patients had 10% and 11%. The extent of the visual fields for patients with esotropia, exotropia and orthotropia are provided in Table 2.

**Table 2:** Visual fields characteristics between groups.

	Esotropes Mean (SD)	Exotropes Mean (SD)	Orthotropes Mean (SD)
Horizontal extent of BVF	122.79 (18.76)	138.25 (8.30)	141.00 (6.60)
Vertical extent of BVF	82.15 (9.33)	83.84 (5.13)	86.00 (0.00)
BVF: points missed	8.00 (8.21)	4.00 (3.25)	1.9 (4.07)
Difference between horizontal extent of the sum of MVFs and the BVF	74.21 (20.71)	68.90 (34.38)	100.60 (2.27)
Amblyopic eye: horizontal extent MVF	98.70 (19.76)	104.75 (16.93)	121.00 (3.16) right eye
Fellow eye: horizontal extent MVF	100.60 (18.45)	102.40 (28.14)	120.60 (3.78) left eye
Amblyopic: vertical extent MVF	75.75 (12.89)	79.74 (12.27)	84.70 (4.11) right eye
Fellow eye: vertical extent MVF	79.90 (9.31)	81.37 (8.96)	84.50 (4.74) left eye
Ratio of HE of Amblyopic eye to BVF	0.79 (0.09)	0.77 (0.12)	0.86 (0.03) right eye
Ratio of HE of fellow eye to BVF	0.81 (0.09)	0.77 (0.11)	0.86 (0.04) left eye
Ratio of HE of BVF to sum of MVF	0.63 (0.06)	0.66 (0.10)	0.58 (0.02)

*BVF* binocular visual field, *MVF* monocular visual field, *HE* horizontal extent.

### Monocular fields

The presence of an esotropia or exotropia were significant factors in the horizontal extent of the visual field of the amblyopic eyes (*p* < 0.01) and fellow eyes (*p* < 0.01). Age (*p* = 0.21, *p* = 0.20), and visual acuity of the fellow or amblyopic eyes (*p* = 0.45, *p* = 0.17) were not significant. There were also no significant associations between the horizontal extent of the visual field (VF) of either the amblyopic or fellow eyes and the refractive errors of patients with esotropia (*p* = 0.44) and exotropia (*p* = 0.39). Similarly, there was no significant association between the horizontal extent of the MVF and visual acuity of the amblyopic (*p* = 0.16 and *p* = 0.66) or fellow eyes (*p* = 0.82 and *p* = 0.43) eyes of patients with esotropia and exotropia, respectively.

**Fig. 1:**
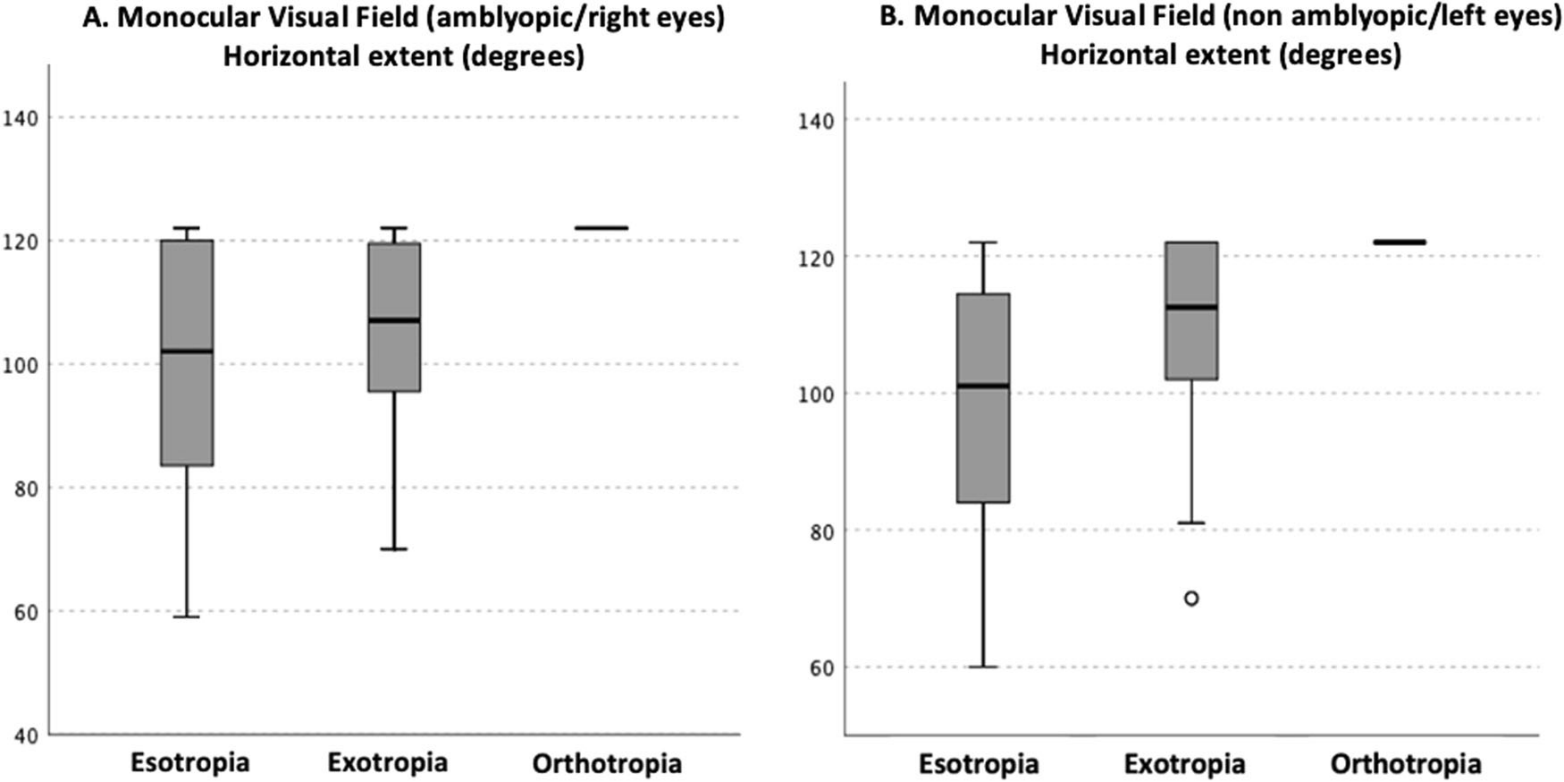
Horizontal extent of the visual field of the amblyopic (**A**) and fellow eye (**B**) eye of patients with esotropia, exotropia and subjects with orthotropia.

The horizontal extent of the VF of the amblyopic eyes of patients with esotropia (98.70 degrees SD 19.76) and exotropia (104.75 degrees SD 16.93) was significantly smaller than eyes with orthotropia (121.00 degrees SD 3.16) by 22.3 degrees (*p* = 0.004) and 16.25 degrees (*p* = 0.045), respectively (Fig. 1A) with higher coefficient of variance (CoV) of 20.02% and 16.59% than that of orthotropes (2.61%). There was no difference in the extent of the monocular horizontal field of the amblyopic eye between the esotropes and exotropes (6.05 degrees *p* = 0.49).

For the fellow eyes, there was a significant difference in the horizontal extent of the VF between patients with esotropia and orthotropia by 20 degrees (p = 0.006) but not between patients with esotropia and exotropia (1.8 degrees *p* = 0.485). There was, however, no difference in the extent of the horizontal VF of the non-amblyopic eye of exotropes and subjects with orthotropia (18.20 degrees *p* = 0.068) (Fig. 1B).

### Binocular visual fields

#### Vertical fields

The sizes of the vertical extent of the BVF are presented in Table 2. The vertical extent of the BVF was not related to the presence of an esotropia or exotropia (*p* = 0.49), age (*p* = 0.43), size of the tropia (*p* = 0.43) or visual acuity of the amblyopic (*p* = 0.86) and fellow (*p* = 0.31) eyes.

#### Horizontal fields

**Fig. 2:**
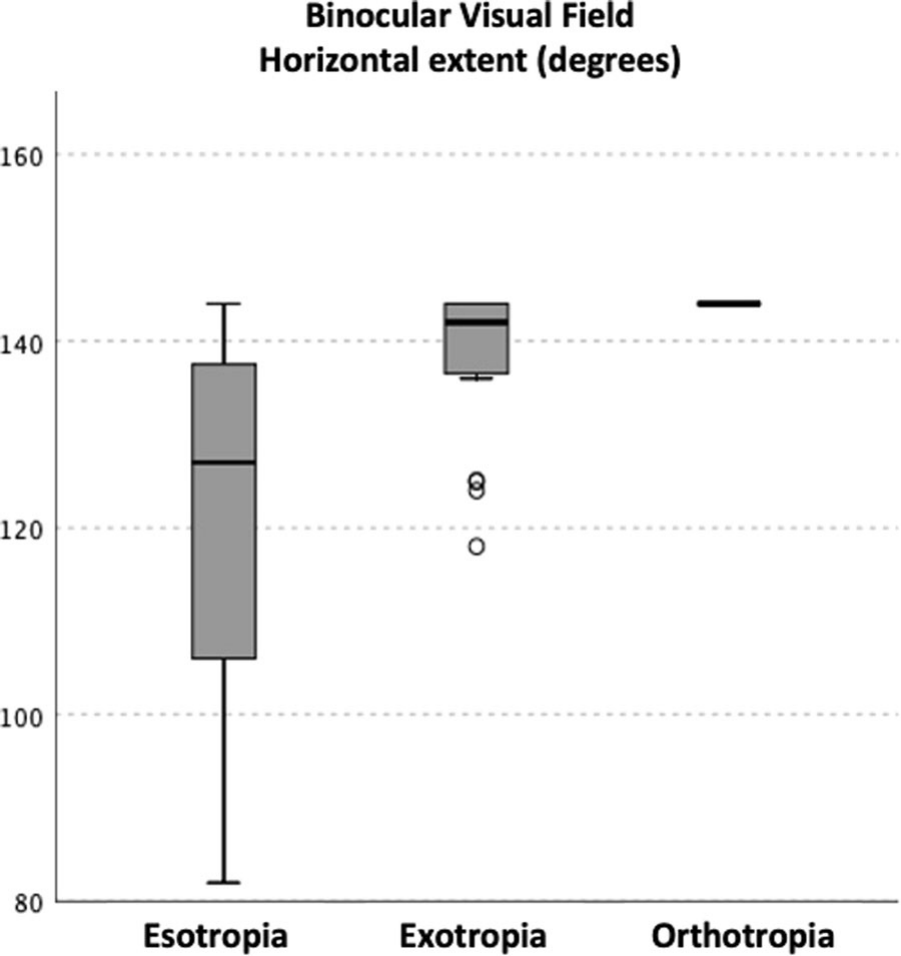
Horizontal extent of binocular visual field in patients with esotropia and exotropia, and subjects with orthotropia.

There were significant differences in the horizontal extent of the BVF in patients with esotropia, exotropia and normal eyes (*p* < 0.01) (Fig. 2).

The presence of a tropia (esotropia or exotropia) was the significant factor in the horizontal extent of the BVF (*p* = 0.005). The difference in the horizontal extent of the BVF between esotropes and orthotropes was −18.21 degrees (*p* = 0.009) and −15.46 degrees for exotropes (*p* = 0.007). There was, however, no significant difference in the horizontal extent of BVF between exotropes and orthotropes (−2.75 degrees p = 0.84). Age (p = 0.21), visual acuity of the amblyopic (*p* = 0.47) and fellow eye (*p* = 0.22), and size of the tropia (*p* = 0.10) were not significant. There was also no significant association between the BVF and the degree of anisometropia for either patients with esotropia or exotropia (*p* = 0.22, *p* = 0.72).

**Fig. 3:**
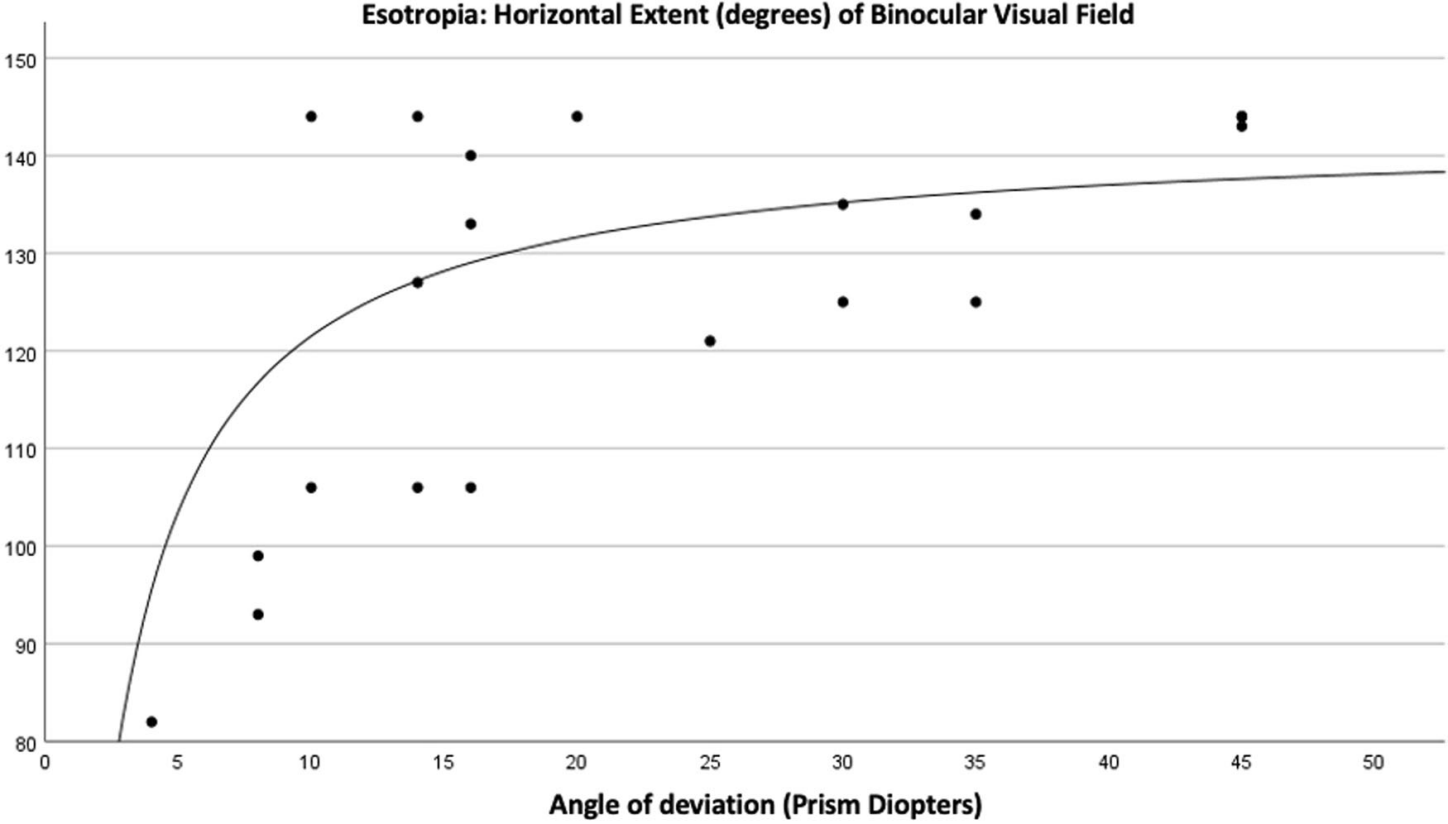
Horizontal extent of the binocular VF and angle of deviation in esotropes. Horizontal extent of VF (HVF) $HVF = e^{4.96 - \frac{{1.60}}{{Angle}}}$.

There was, however, a significant non-linear association between the horizontal extent of the BVF of patients with esotropia and the angle of deviation (R^2^ = 0.63, *p* = 0.005) (Fig. 3) but not for those with exotropia (*p* = 0.45). Horizontal extent of VF (HVF) $HVF = e^{4.96 - \frac{{1.60}}{{Angle}}}$.

The ratio of the horizontal extent of the BVF to the sum of the MVF was related to the size of the tropia (*p* = 0.017), VA of the fellow (non-amblyopic) eye (*p* = 0.016) and the diagnosis (esotropia, exotropia or orthotropia) (*p* = 0.014), but not the VA of the amblyopic eye (*p* = 0.98). The ratio of the BVF to the sum of the MVF was similar between esotropes and exotropes (*p* = 0.88), but smaller in orthotropes (0.58) compared to either esotropes (0.63, *p* < 0.01) or exotropes (0.66, *p* < 0.01) (Supplementary Fig. 1).

The ratio of the horizontal extent of the VF of the amblyopic to non-amblyopic eye or the ratio of non-amblyopic eye to binocular field was not associated with the difference in VA between the amblyopic and fellow eyes (*p* = 0.21 and *p* = 0.81). Similarly, there was no significant difference in the ratio of the horizontal extent of the VF of amblyopic to fellow eyes between esotropes and orthotropes or exotropes (*p* = 0.97, *p* = 0.96) or between exotropes and subjects with orthotropia (*p* = 0.99). There was no difference in the ratio of the horizontal extent of the BVF on the side of amblyopic/right eye to fellow eye side (*p* = 0.31) (Supplementary Fig. 2).

The HE of BVF on the amblyopic side was significantly smaller for esotropes (*p* = 0.004) compared to exotropes (*p* = 0.008) and orthotropes (*p* = 0.003). There was no difference between exotropes and orthotropes (*p* = 0.45).

### Points missed in the visual field

There were more points missed for the amblyopic eyes of patients with esotropia 9.85 (8.73) than those with orthotropia 1.30 (2.16) (*p* = 0.003) but not compared to those with exotropia 7.37 (6.78) (*p* = 0.58). There was also a significant difference between exotropes and orthotropes (*p* = 0.02).

There were more points missed in the BVF for the amblyopic eyes of patients with esotropia 8.00 (8.21) than those with orthotropia 1.90 (4.01) (*p* = 0.02), but not exotropia 4.00 (3.25) (p=0.44). There was no difference between exotropes and orthotropes (*p* = 0.40).

In 31.6% (6 of 19) of the patients with esotropia, the horizontal extent of their binocular field was below 120 degrees and as such would not meet the driving standard. These patients had a smaller angle of deviation (mean 11PD, min 4PD, max 16PD) than those whose binocular field was >120 degrees (mean 25PD, min 10PD, max 45PD), (*p* = 0.002). There were no differences in either near or distance visual acuity (*p* = 0.78, *p* = 0.55) between them. No patients with exotropia and no subjects with orthotropia had a horizontal BVF of less than 120 degrees.

## Discussion

The horizontal extent of the BVF of esotropes, was 18.2 degrees or 21% smaller than that of orthotropes and also more variable compared to exotropes and orthotropes. This is similar to that reported by Quah and Kaye who reported a difference between children with esotropia and orthotropia of 19 degrees (138 for orthotropes and 119 for esotropes) using a Goldmann visual field. In addition, we found that there were significantly more points missed in amblyopic, fellow, and binocular fields of esotropes compared to orthotropes.

The relationship between the size of the esotropia and BVF may indicate increased suppression of the peripheral binocular field with reducing size of the esotropia. Quah and Kaye reported a significantly smaller BVF to summed MVF ratio in children with esotropia compared to orthotropia of 0.56 vs. 0.59. In contrast, although we found a similar ratio in orthotropes (0.58), increased ratios of the BVF to summed MVF in esotropes (0.63) and exotropes (0.66) in adults were observed. This increased BVF to summed MVF ratio in esotropes and exotropes may reflect the smaller MVF we found for both the amblyopic and fellow eye of esotropes and exotropes compared to orthotropes.

The absence of a difference in the horizonal extent of the visual field between right and left eyes of orthotropes or between amblyopic and fellow eyes of esotropes or exotropes would suggest that the BVF is symmetrical between eyes. The contribution of the monocular field of the fellow eye to the binocular field of esotropes was significantly greater than that in subjects with orthotropia. This suggests a greater dependency of the horizontal extent of the BVF on that of the fellow eyes of patients with esotropia compared to orthotropes.

In 31.6% (6 of 19) of the patients with esotropia, the horizontal extent of their BVF was below 120 degrees and as such would not for example, meet the driving standard in the UK. These patients also had a smaller angle of deviation than those whose binocular field was >120 degrees (25PD). In the United Kingdom, the DVLA have established national medical guidelines on fitness to drive which can be accessed online [15]. With regard to the visual field, the minimum field of vision for Group 1 driving (Car and Motorcycle) is defined in the legislation: “A field of at least 120° on the horizontal measured using a target equivalent to the white Goldmann III4e settings. The BVF should extend at least 50° to the left and right. In addition, there should be no significant defect in the binocular field that encroaches within 20° of the fixation above or below the horizontal meridian”. Higher standards of field of vision are required for Group 2 (Bus and Lorry): “an uninterrupted measurement of at least 160° on the horizontal plane, extensions of at least 70° left and at least 70° right, extensions of at least 30° above and at least 30° below the horizontal plane, no significant defect within 70° left and 70° right between 30° up and 30° down, no defect is present within a radius of the central 30°, and no other impairment of visual function, including no glare sensitivity, contrast sensitivity or impairment of twilight vision.” [1] More details regarding other requirements and defects affecting central area can be accessed [1, 15] A recent small study using driving simulator in France found significant improvement in Esterman Visual Field score and self-confidence during driving after strabismus surgery [16].

Visual field requirements for driving licence in The European Union are similar to the UK [17]. Van Rijn et al. estimated the prevalence of visual field defect in the European drivers to be between 0.5 and 0.8% in the age group 45-74 years old and 2.7% in the age group ≥75 years old, in the 2373 drivers, visual field defects did not interfere with driving capacity in the majority of glaucoma patients (2/39) [18]. It is not known, however, how many of these drivers had horizontal strabismus. All Canadian jurisdictions except Quebec have standards of horizontal extent of BVF of 120 degrees. In the US, not all jurisdictions have specific visual field standards, only 9 states have the minimum of BVF horizontal extent of 120 degrees [19]. This shows that there is no worldwide agreement on the minimum standards of the field of vision for driving which could be partly due to the limited research about the influence of vision and extent of BVF on driving. Therefore, there is a need for more studies and research to provide valid information to policymakers.

The main limitation of this study is that although all visual field tests were done by the same technician, we did not repeat the visual fields to assess the repeatability of the extent of the BVF. The VF, however, were reliable with few false negatives and positives. We did not examine if the BVF in subjects with an abnormal head posture (AHP) would have changed if they were allowed to maintain AHP during the VF test. Similar studies should be undertaken in other centres to determine if these findings are generally applicable across populations.

In this study we have reported the extent of BVF in adults with esotropia, exotropia and subjects with no strabismus using Humphrey Esterman visual field. We found that the horizontal extent of BVF is significantly smaller and more variable in adults with congenital esotropia. Adults with esotropia applying for driving license may fall below visual field standard in the UK and in Europe. This would suggest that people with an esotropia should be offered the opportunity have a visual field test specifically a BVF test before applying for a driving license. Further future studies are needed to determine and investigate this in other populations.

## Summary

### What was known before

- If a person has a strabismus (misalignment of their eyes) but does not have diplopia and has a corrected visual acuity of at least 6/12, then a BVF test is not required for driving a car.

### What this study adds

- We found that the horizontal extent of the binocular visual field is significantly smaller and more variable in adults with esotropia and that in 31.6% of cases, was below the driving standard. Adults with an esotropia, therefore, may fall below visual field standard in the UK and in Europe.

## Supplementary information

Supplementary Figure 1

Supplementary Figure 2

Supplementary Figure Captions

## Data Availability

Data available on request.
