# Supplementary figures and images for: Binocular visual field in adults with horizontal strabismus and driving requirements
Source: Eye (Lond). 2022 Dec 2;37(11):2220–5. doi: 10.1038/s41433-022-02319-5 (PMC10366138; doi:10.1038/s41433-022-02319-5)

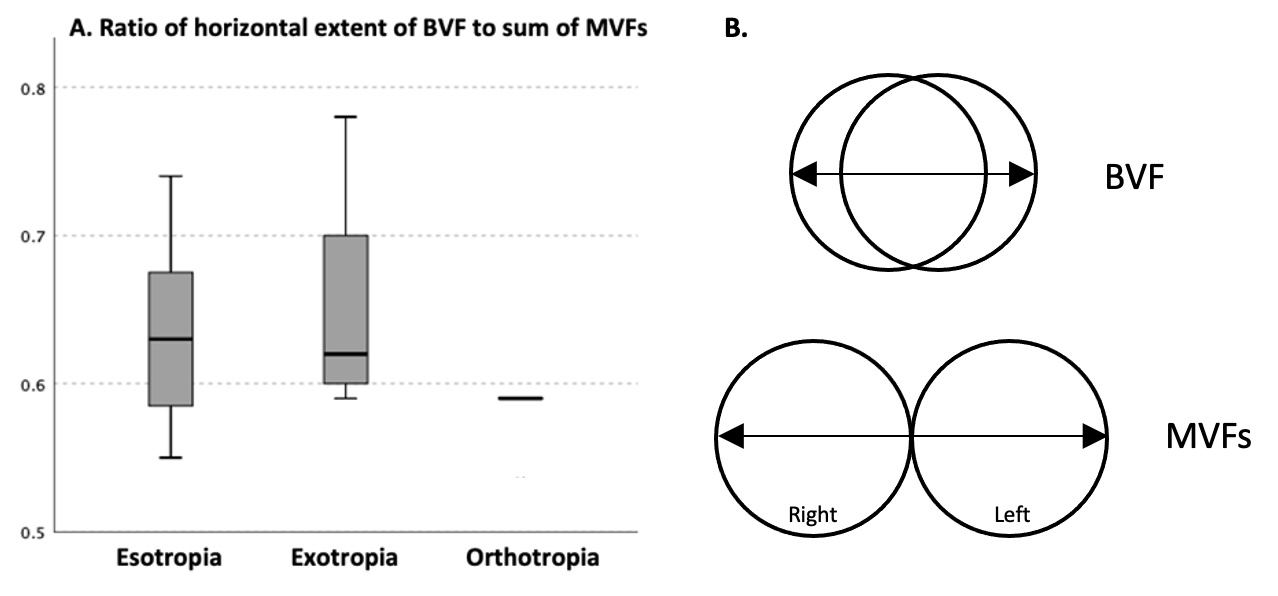

Supplement: Supplementary file 1 — Supplementary Figure 1 [file 41433_2022_2319_MOESM1_ESM.jpg]

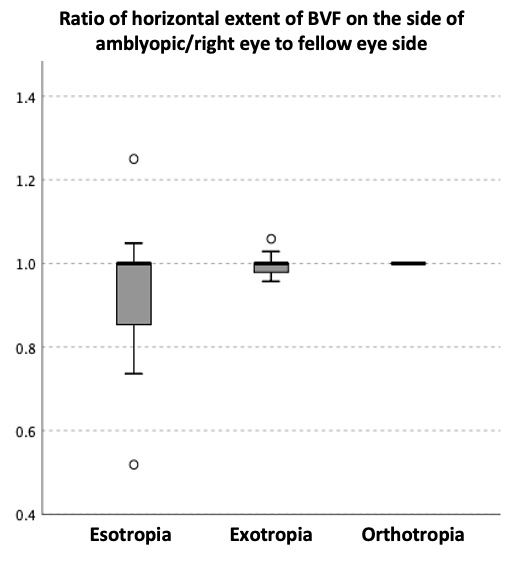

Supplement: Supplementary file 2 — Supplementary Figure 2 [file 41433_2022_2319_MOESM2_ESM.jpg]
