# Supplementary material for: Binocular visual field in adults with horizontal strabismus and driving requirements
Source: Eye (Lond). 2022 Dec 2;37(11):2220–5. doi: 10.1038/s41433-022-02319-5 (PMC10366138; doi:10.1038/s41433-022-02319-5)
Supplement: Supplementary file 3 — Supplementary Figure Captions [file 41433_2022_2319_MOESM3_ESM.docx]

**Supplementary Figure 1.** Ratio of the horizontal extent of the BVF to the sum of the MVFs in patients with esotropia, exotropia and subjects with orthotropia (A). Representative diagram of BVF and summed MVFs (B).

**Supplementary Figure 2.** Ratio of the horizontal extent of the BVF on the side of amblyopic/right eye to fellow eye side
